# Rapidly Fatal Pulmonary Mucormycosis With Pericardial Dissemination: A Case Report and Imaging Insights

**DOI:** 10.1155/crdi/3636062

**Published:** 2025-10-21

**Authors:** Zahra F. Rahmatullah, Satomi Kawamoto, Elliot K. Fishman

**Affiliations:** Russell H. Morgan Department of Radiology and Radiological Science, Johns Hopkins University School of Medicine, 600 N Wolfe St, Baltimore 21287, Maryland, USA

**Keywords:** CT, disseminated mucormycosis, immunocompromised, mucormycosis, pericardial mucormycosis, pulmonary angioinvasion

## Abstract

Mucormycosis is a rare but aggressive opportunistic fungal infection, predominantly affecting immunocompromised individuals. We report a case of a 59-year-old male with newly diagnosed acute myeloid leukemia undergoing chemotherapy who developed pulmonary mucormycosis, which rapidly progressed to disseminated disease with pericardial involvement, an exceptionally rare occurrence. Initial chest CT imaging showed a subtle perihilar infiltrate, but within days, extensive spread was evident, showing widespread pulmonary consolidation, ground-glass opacities, vascular thrombosis, mediastinal invasion, and pericardial involvement. Bronchoscopy confirmed airway obstruction due to fungal invasion, and biopsy, along with pericardial fluid cultures, revealed *Rhizopus microsporus*. Despite early antifungal treatment, the patient's condition worsened, making surgery impossible and leading to respiratory failure and death. This case underscores the rapid progression and extensive spread of mucormycosis, highlighting the critical role of early CT imaging and clinical vigilance in high-risk patients. Timely recognition and intervention are essential to improve outcomes through both early medical and possible surgical management in this often-fatal disease.

## 1. Introduction

*Rhizopus microsporus,* a filamentous fungus of the *Mucoralean* genus, causes rare and often fatal infections referred to as mucormycosis or zygomycosis [1]. The clinical manifestations are classified based on the presentation, which can be rhinocerebral, pulmonary, cutaneous, gastrointestinal, disseminated, or rare manifestations such as cardiac or pericardial involvement [2, 3]. Mucormycosis is mostly reported in immunocompromised individuals such as those with diabetes mellitus, hematological malignancies, solid organ transplants, or corticosteroid use [4, 5]. It is characterized by angioinvasion and is associated with a high morbidity and mortality [6]. In this article, we report a unique case of a 59-year-old male with pulmonary mucormycosis who had a seemingly mild clinical presentation initially, which rapidly progressed into disseminated disease with pericardial involvement and corresponding extraordinary computed tomography (CT) findings.

## 2. Case Presentation

**Figure 1:**
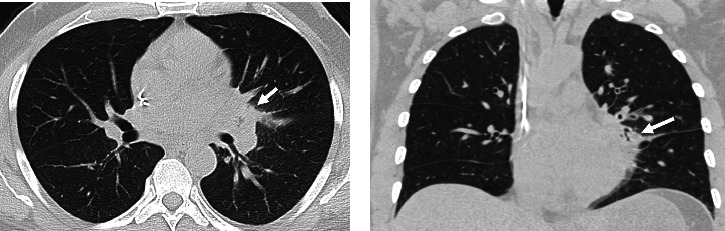
A 59-year-old male with AML undergoing chemotherapy developed fever, cough, and pleuritic chest pain. CT without contrast axial view (a) and coronal view of the chest in the lung window (b) show early infiltrate in the left perihilar region (white arrow).

A 59-year-old male presented to our institution as he was incidentally found to be pancytopenic on laboratory tests. He was diagnosed with acute myeloid leukemia (AML) and was admitted for treatment with a “7 + 3” chemotherapy regimen (cytarabine + daunorubicin) while also being enrolled in a clinical trial using ziftomenib. During chemotherapy, he developed a fever resistant to multiple antibiotics, and all blood cultures were negative for bacterial infections. This was accompanied by a cough and pleuritic chest pain, and a CT scan of the chest without IV contrast (Figure 1) at the time of initial symptoms showed a subtle infiltrate in the left perihilar region.

**Figure 2:**
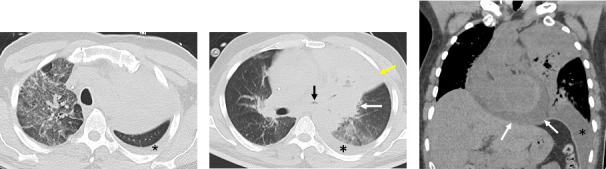
CT without contrast axial views 7 days later (a, b) in the lung window show worsening of left upper lobe consolidation (yellow arrow) and left perihilar consolidation (white arrow), diffuse narrowing of the left bronchi with heterogenous intraluminal material (black arrow), and right upper lobe and left lower lobe ground-glass opacities (reverse halo sign). Left pleural effusion is visualized (asterisk). Coronal soft tissue window view (c) shows a new pericardial effusion (white arrows) secondary to dissemination to the pericardium.

While on extensive antibiotic treatment, the patient developed a new oxygen requirement and worsening dyspnea. Seven days after the initial CT, follow-up CT without IV contrast (Figure 2) was obtained for worsening dyspnea, which showed increased pulmonary consolidation, ground-glass opacities, worsening of left bronchial narrowing with intraluminal material, and new moderate pericardial effusion measuring up to 2 cm in thickness. On the following day, the patient developed chest pain and tightness with elevated serum troponin, and an electrocardiogram demonstrated diffuse ST-segment elevations with PR depressions. After being transferred to the intensive care unit, a transthoracic echocardiogram showed a large pericardial effusion measuring up to 3 cm in thickness. Left ventricular ejection fraction was estimated at 70%, with no regional wall motion abnormalities. The patient was clinically diagnosed with myopericarditis, which was initially thought to be related to cancer treatment agents. A pericardial drain was then placed to achieve hemodynamic stability, and the fluid was sent for culture. The next day, the patient clinically deteriorated and developed acute hypoxemic respiratory failure for which he was intubated. A bronchoscopy was performed to obtain cultures, and a chest tube was placed for a large left-sided pleural effusion.

**Figure 3:**
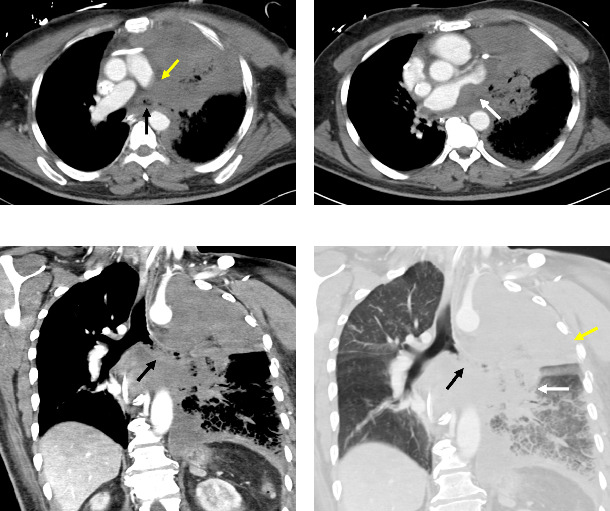
A 59-year-old male with AML undergoing chemotherapy developed fever, cough, pleuritic chest pain, and eventual respiratory failure requiring intubation due to pulmonary mucormycosis that evolved into disseminated disease. Axial views of CT chest images with IV contrast in soft tissue window 11 days later (a and b) show persistent consolidation of the left lung with left main stem bronchus occlusion (black arrow) and complete thrombosis of the left main pulmonary artery (yellow arrow) with absent pulmonary arterial flow to the left lung. There is soft tissue infiltration in the mediastinum with mass effect on the left atrium (white arrow). Coronal views in (c) soft tissue window and (d) lung window show occlusion of the left main stem bronchus with heterogeneous intraluminal material (black arrow) with complete left upper lobe consolidation (yellow arrow) and left perihilar consolidation (white arrow) with diffuse ground-grass opacity in the left lower lobe.

Eleven days after his initial chest CT, the patient underwent CT with intravenous contrast (Figure 3), which showed persistent consolidation and ground-glass opacities in the left lung, and persistent multifocal areas of ground-glass opacities in the right lung, representing multifocal pneumonia. Soft tissue infiltration in the mediastinum near the left hilum was identified with mass effect on the left atrium. There was also complete occlusion of the left main stem bronchus with heterogeneous intraluminal material and occlusive thrombosis of the left main pulmonary artery with absent pulmonary arterial flow to the left lung. Left pleural effusion and pericardial effusion were decreased due to the placement of a pericardial drain and left chest tube.

**Figure 4:**
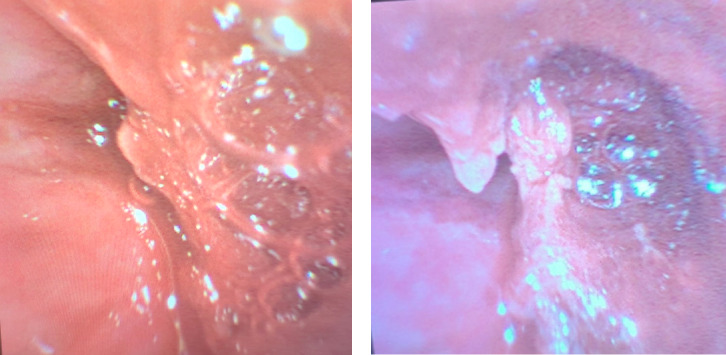
A 59-year-old male with AML undergoing chemotherapy developed respiratory failure due to pulmonary mucormycosis that evolved into disseminated disease. Bronchoscopy images (a, b) reveal an obstructed left mainstem bronchus, with tissue resembling mucosa and some surrounding granulation.

On bronchoscopy, the CT findings were corroborated, as the patient had an obstruction of his left main stem bronchus with tissue that appeared similar to his mucosa, though with some surrounding granulation (Figure 4). On biopsy, numerous fungal hyphae were found involving necrotic tissue with angioinvasion. Cultures from pericardial effusion and bronchoalveolar lavage (BAL) subsequently grew *Rhizopus microsporus*. Quadruple antifungals (IV and inhaled amphotericin B liposome, micafungin, and isavuconazole) were started along with bacterial and herpes virus prophylaxis, but the patient remained intubated and critically ill due to the invasive mucormycosis. Further bronchoscopy for debulking of the obstructing mycetoma was conducted; however, there remained evidence of invasion into all of the left-sided central airways, and the patient was not stable enough to undergo thoracic surgery, given the extensive involvement of the disease. The patient was transferred to palliative care and passed away after extubation.

## 3. Discussion

*Rhizopus microsporus* is a filamentous fungus classified within the Mucorales order. While species from the *Rhizopus* genus are used in food preparation, they can also cause mucormycosis, a rare angioinvasive opportunistic infection [1]. The most common route of infection is through inhalation of spores residing in the soil or organic matter [7]. Mucormycosis was previously thought to only affect immunocompromised patients; however, recent data have shown that immunocompetent individuals can also develop this infection [8]. The incidence is unknown, as most data stem from case reports and small case series that are often population-specific. However, recent data trends suggest that the incidence is on the rise and mucormycosis is considered an emerging disease worldwide [9]. A single-center study in India from 1990 to 2007 described the incidence of mucormycosis to be 12.9 cases/year over the first decade and increased to 35.6 cases/year over a 5-year period after that, and most recently, during an 18-month period, reached 50 cases/year [10–12]. Depending on the clinical presentation, mucormycosis is classified as rhinocerebral, pulmonary, cutaneous, gastrointestinal, disseminated, or other atypical manifestations such as endocarditis, pericarditis, osteomyelitis, peritonitis, renal involvement, and isolated cerebral infections [9]. Disseminated disease, involving two or more organ systems, often leads to the most critical illness, with mortality increasing up to 96%, and is associated with extreme immunosuppression [2, 13].

Previous studies highlighted fever, dyspnea, and cough as the most common initial clinical symptoms in pulmonary mucormycosis [14]. Our patient had a fever, severe dyspnea, and later also demonstrated thrombosis of the pulmonary vasculature as well as pericardial involvement due to progression of pulmonary mucormycosis evolving to disseminated disease. Only a small number of case reports documented cardiac involvement in disseminated mucormycosis originating from primary pulmonary foci. However, it typically occurs in patients with predisposing conditions that lead to disseminated disease or as a complication following cardiac surgery [15]. The cardiac manifestations of mucormycosis include myocarditis, myocardial infarction resulting from fungal invasion of vessel walls, endocardial invasion from adjacent infected thrombus or myocardium, valvular vegetations leading to incompetence, conduction system complications, and pericardial involvement [16–18]. Pericardial involvement includes forms of pericarditis and pericardial effusion, though these are rarely reported due to their infrequency. Symptoms such as characteristic chest pain, pericardial friction rub, and atrial fibrillation have been observed, with one case even progressing to refractory cardiac tamponade [19]. While incredibly rare, a high degree of clinical suspicion is essential for timely intervention to avoid rapid clinical decline, as observed in our patient.

Homogenous consolidations have been documented as the most common abnormality on chest imaging with additional findings including nodules, ground-glass opacities, cavitation occasionally with an air crescent sign (gas between the cavitary wall and intracavitary mass), necrosis, and pleural effusion [20]. Rarely, endobronchial involvement can also be seen, manifesting as invasive endobronchial or endotracheal mass [21]. While some of these findings can be appreciated on chest radiographs, CT is the preferred modality as it can pick up on finer details and any vessel or endobronchial involvement which are commonly seen in mucormycosis [22]. The reverse halo sign is also commonly found in pulmonary mucormycosis and is defined as a ground-glass lesion with a peripheral rim of consolidation [21, 23]. In addition, the reverse halo sign serves as a useful radiologic feature to help differentiate mucormycosis from invasive pulmonary aspergillosis, a clinically similar fungal infection. One study found that most patients with pulmonary mucormycosis, at some point of the disease course, had an identifiable reverse halo sign in 67%, ground-glass opacities larger than the lesion in 53%, peripheral predominance of lesions in 87%, pulmonary artery thromboembolism in 20%, mediastinal invasion in 6%, and endobronchial involvement in 3% on CT [22]. Furthermore, in 17% of patients, CT revealed a multifocal pneumonia pattern, and these patients were associated with higher disease severity and mortality rate [24]. This is noteworthy, as our patient initially presented with only a focal infiltrate in the left lung (Figure 1). However, just 7 days later, follow-up CT imaging revealed large consolidation with reversed halo sign, diffuse narrowing of the left bronchi, pleural effusion, and pericardial dissemination, which is significantly more aggressive and extensive disease progression than is typically observed (Figure 2).

Definitive diagnosis is based on microscopy using optical staining such as Blankophor or Calcofluor White on histopathology and culture specimens [22, 25]. A polymerase chain reaction (PCR)–based diagnosis of pulmonary mucormycosis has been developed with encouraging results. A study compared BAL fluid and serum quantitative PCR (qPCR) as diagnostic tools and found that BAL qPCR was only positive in 50% of patients, while serum qPCR was positive in 79% of patients and associated with an earlier diagnosis [3]. Due to the rapid progression of the infection, early diagnosis is pivotal to promptly start treatment in order to halt or limit invasion and improve patient prognosis. Treatment involves a comprehensive strategy including correction or withdrawal of underlying risk factors when possible, early administration of antifungal medications, surgical removal of all infected tissues, and the use of various adjunctive therapies such as hyperbaric oxygen and granulocyte colony–stimulating factor (G-CSF) [25]. First-line therapy consists of liposomal amphotericin B, combined with surgery whenever possible, while isavuconazole and posaconazole are considered second-line agents [26]. Timely treatment is essential; one study found that delaying amphotericin B-based therapy by more than five days in patients with mucormycosis and underlying hematological malignancies resulted in an approximately twofold increase in 12-week mortality [27].

## 4. Conclusion

Mucormycosis, although relatively rare, poses a significant threat to immunocompromised patients due to its persistently high mortality. Diagnosis and treatment of mucormycosis remain challenging, and we highlight an unusual presentation of the disease with rapid progression from pulmonary mucormycosis to disseminated disease with pericardial involvement. This case highlights the role of serial CT imaging in detecting and monitoring the progression of the disease and the role of characteristic CT findings in differentiating mucormycosis from other pulmonary infections. Given the aggressive nature of the disease, clinicians must maintain a high index of suspicion in patients with risk factors, particularly those who are immunocompromised. Early recognition of clinical deterioration and associated radiologic patterns can guide timely medical therapy, surgical management, and adjunctive therapies such as hyperbaric oxygen and G-CSF, ultimately improving patient outcomes.

## Data Availability

Data sharing is not applicable to this article as no new data were created or analyzed in this study.
